# A Case of Trousseau's Syndrome Accompanying Ovarian Cancer with Widespread Thromboembolisms

**DOI:** 10.1155/2020/3738618

**Published:** 2020-06-05

**Authors:** Hiroharu Kobayashi, Yoshifumi Arai, Kentaro Iga, Misa Kobayashi, Takashi Suzuki, Satoru Nakayama, Hiroshi Adachi

**Affiliations:** ^1^Department of Gynecology, Seirei Hamamatsu General Hospital, Japan; ^2^Department of Pathology, Seirei Hamamatsu General Hospital, Japan

## Abstract

The patient was a 41-year-old woman, gravida 0. She had no notable medical history. Laparoscopic right salpingo-oophorectomy and left cystectomy were performed for bilateral ovarian endometriomas, which were both pathologically diagnosed as benign. Six months later, she presented with left lower abdominal pain and expressive aphasia. Examination revealed multiple cerebral infarctions and pulmonary embolism. The patient was diagnosed with Trousseau's syndrome secondary to ovarian cancer, and anticoagulant therapy was initiated. Despite treatment, she developed visual field loss due to occlusion of the left retinal artery; dizziness due to cerebellar infarction and myocardial infarction; and right hemiplegia due to new cerebral infarction. She received chemotherapy (two courses of paclitaxel and carboplatin), which did not improve her condition, and died two months after onset. An autopsy revealed that her left ovary was enlarged to a size of 12 cm and an endometrioid carcinoma G2 was identified. Ovarian cancer had spread throughout the abdominal cavity, and a large amount of pleural and ascites fluid was present. Multiple thrombi were found in bilateral pulmonary arteries and bilateral common iliac veins. There was a 2.5 cm thrombus in the left ventricle apex, and the anterior descending branch was obstructed by thrombus with recanalization.

## 1. Introduction

In 1865, Trousseau reported a case of cerebral infarction and pulmonary embolism due to multiple venous thrombosis in a patient with gastric cancer [1]. Cancer patients may have an abnormality of coagulation and fibrinolysis [2]. This pathological condition often causes cerebral infarction, and is called “Trousseau's syndrome.” We experienced a case of Trousseau's syndrome with widespread emboli (multiple cerebral infarctions, cerebellar infarction, pulmonary embolism, myocardial infarction, occlusion of retinal artery, and kidney infarction) in a patient with ovarian cancer. Most of the reports of Trousseau's syndrome involve cerebral infarction. Although there were reports of myocardial infarction, there has been no report of Trousseau's syndrome with such widespread thromboembolic symptoms.

## 2. Case Presentation

The patient was a 41-year-old woman, gravida 0. She had notable medical history. Laparoscopic right salpingo-oophorectomy and left cystectomy were performed for bilateral ovarian endometriomas, which were both pathologically diagnosed as benign. After surgery, she underwent regular outpatient treatment with dienogest. Six months later, she presented with left lower abdominal pain and expressive aphasia. A 7 cm diameter cyst had formed in the left ovary, with a solid ingredient and surrounding ascites. Contrast-enhanced computed tomography (CECT) revealed peritoneal dissemination, multiple lymph node metastases, liver metastasis, pulmonary embolism, and deep venous thrombosis of the lower limbs. Brain magnetic resonance imaging (MRI) revealed that infarctions occurred at multiple locations in the bilateral cerebral hemisphere centered on the left frontal lobe and in the right cerebellar hemisphere. She was diagnosed with advanced ovarian cancer, accompanied by Trousseau's syndrome. She was hospitalized (Day 0) and received 10,000 units of heparin per day intravenously. On Day 11, she developed a left visual field deficit due to occlusion of the left retinal arterial bifurcation. On Day 17, she had vertigo and vomiting. Brain MRI showed new infarctions of the cerebellar vermis, left caudate nucleus head, and cerebral subcortex. On Day 26, the first chemotherapy was administered (paclitaxel 175 mg/m^2^ and carboplatin AUC5). On Day 29, a blood transfusion was performed for anemia due to digestive tract bleeding. On Day 30, she had chest pain. Myocardial infarction was suspected from ST elevation on electrocardiogram and hypokinesis from the anterior wall to the apex on echocardiography. Stent treatment must be combined with antiplatelet therapy, which could not be performed with the administration of heparin in the presence of gastrointestinal bleeding. Instead, she was administered an increased dose of heparin (20000 units per day) and isosorbide dinitrate. On Day 35, paralysis of the right upper and lower limbs appeared. Brain MRI showed occlusion of the left middle cerebral artery. On Day 49, the second chemotherapy was administered (paclitaxel 175 mg and carboplatin AUC5). On Day 66, she died due to multiple organ failure with no improvement of thrombotic symptoms.

**Figure 1:**
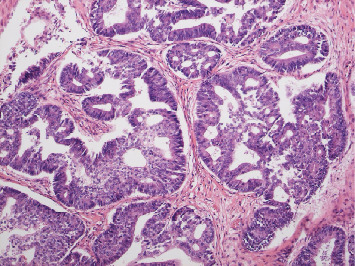
Adenocarcinomas are arranged in irregularly glandular and tubular or cribriform pattern, showing morphology of endometrioid carcinoma. Hematoxylin and eosin staining x100.

**Figure 2:**
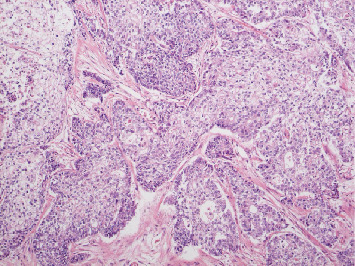
Adenocarcinoma partially shows squamous metaplasia, and components that developed in a sheet-like form are shifting to increased grade of endometrioid carcinoma. Hematoxylin and eosin staining x100.

**Figure 3:**
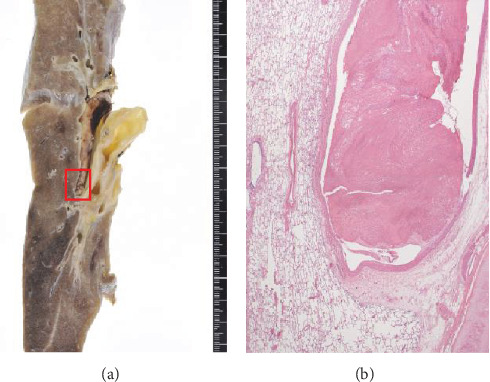
Pulmonary embolism at the hilar portion of (a) lung. (b) is an enlarged image of a pulmonary embolism (x20).

**Figure 4:**
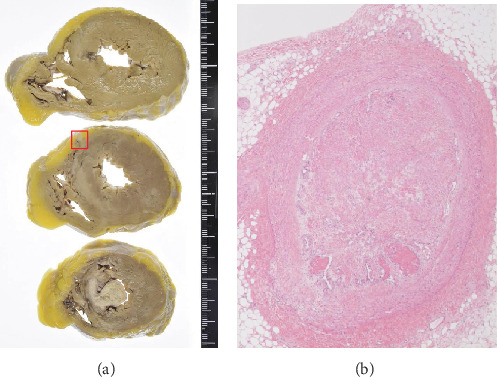
The heart has myocardial infarction from the septum to the lower wall of the (a) ventricle. There is a mural thrombus in the lumen at the apex. The red frame is the anterior descending branch, in which obstruction and recanalization with organization due to thromboembolism are seen ((b) x40).

**Figure 5:**
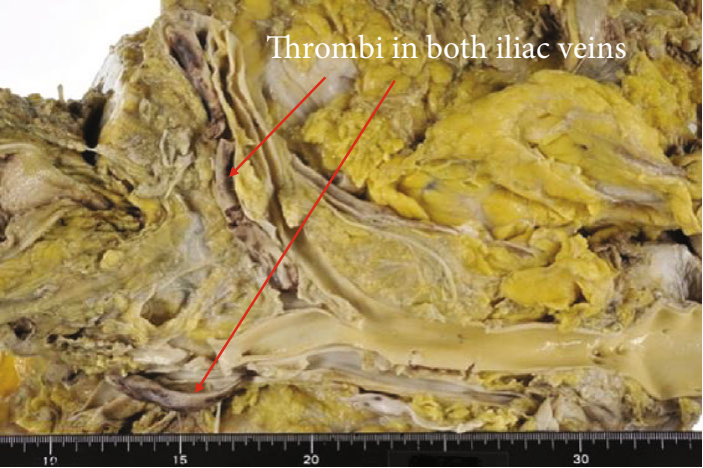
Both iliac veins were filled with organized thrombi.

An autopsy was performed. The results were as follows. A 12 × 8 cm solid mass was found in the left ovary, and it was identified as an endometrioid carcinoma G2 (Figures 1 and 2). Ovarian cancer directly invaded the uterus, bladder, rectum, sigmoid colon, small intestine, retroperitoneum, and left ureter. Metastases were found in the liver, spleen, lung, retroperitoneal, and perigastric lymph nodes. Severe cancerous peritonitis and cancerous pleurisy were found with bloody ascites (4000 ml) and bloody pleural effusion (left 400 ml, right 1000 ml). There was also the presence of a pulmonary embolism; a fibrin thrombus occluded the pulmonary artery in the left hilar region and a small thrombus in part of the right pulmonary artery (Figure 3). In the heart, a 2.5 cm old chronic infarction with massive thrombus in the left ventricular apex was identified, and thromboembolism and recanalization in the anterior descending artery were noted (Figure 4). In relation to the kidney, infarct in 2 × 1 cm size at the lower pole of the right kidney was present. There were three venous thromboses identified: 11 cm long and 9 cm long fibrin clots filling the left and right common iliac veins, respectively, and there was also a 5 mm diameter thrombus in the portal vein (Figure 5). Multiple paraneoplastic thrombosis, in addition to severe cancerous peritonitis and pleurisy with abundant pleural effusion and ascites due to the development of ovarian cancer, leads to patient death.

## 3. Discussion

The mechanism by which malignant tumors cause hypercoagulability is not yet fully understood. Mucin, tissue factor, and cysteine proteinase were reported as possible substances released from tumors to enhance coagulation [3–5]. Patients with ovarian cancer often have thromboembolism, and many, from a histological standpoint, are clear cell carcinomas, which express a large amount of tissue factor [6, 7]. The tumor in the blood vessel itself may flow and embolize the blood vessel elsewhere [8]. It is known that D-dimer increases in the blood of patients with Trousseau's syndrome [9]. In this case, D-dimer showed elevated levels (58.3 *μ*g/ml). CA19-9, a marker for ovarian mucinous tumors, also showed abnormally elevated levels (54,206 U/ml). There are also several reports that mucin produced from the mucinous tumor is associated with thrombus [10–12].

In Trousseau's syndrome, a venous thrombus is formed in the lower limbs and heart due to hypercoagulability caused by tumors, and this is thought to cause multiple emboli in the lung, coronary arteries, and brain. Vein thrombus in the lower limbs in a patient with a patent foramen ovale or vegetation attached to valves in a patient of nonbacterial endocarditis can cause cerebral infarction. However, nonembolic (i.e., in-situ thrombotic) cerebral infarction can also occur in the patient of cancer-related thromboembolism [2, 13]. Hypercoagulable state with in-situ coronary thrombosis can cause myocardial infarction in a patient with ovarian cancer and without thrombotic risk [14]. This is a case in which a thrombus is formed directly in an artery due to hypercoagulability, and patients may not always have a predisposition like atherosclerosis. In this case, embolism of the pulmonary artery must have been caused by the flow of intrailiac thrombi. The patient had no vegetation in the valve, and there was no finding of nonbacterial thrombotic endocarditis. However, she had a mural thrombus in the apex of the left ventricle. It is clinically unclear whether it was caused by the onset of the myocardial infarction or whether it was already present at the time of the first onset of cerebral infarction. The autopsy revealed that the patient did not have a patent foramen ovale. Therefore, intrailiac thrombus was unlikely to cause cerebral or myocardial infarction. Although the patient had no arrhythmia such as atrial fibrillation, failure of myocardial contraction due to myocardial infarction may have caused a thrombus to form in the left ventricle. If so, no ventricular thrombus will have formed at the time of the first onset of cerebral infarction, and the cerebral and myocardial infarction are thought to have occurred due to thrombi that were formed directly in local arteries by hypercoagulability. Alternatively, a thrombus may have been formed in the left ventricle due to hypercoagulability, which may have migrated and occluded arteries to causemultiple cerebral and myocardial infarctions. The lesion in the coronary artery had already been organized and repenetrated over time, and it was impossible to distinguish whether it was a thrombus or embolus. Atherosclerosis was not found in the coronary arteries.

Heparin is effective in acute treatment and prophylaxis for thromboembolism in Trousseau's syndrome [15–18]. If Trousseau's syndrome is caused by venous thrombosis and subsequent embolism, heparin will be effective. Low molecular weight heparin causes less major bleeding complications compared with unfractionated heparin [19]. Warfarin has been reported to be less effective at preventing recurrence of stroke compared to heparin, for unknown reasons [20]. Although there is no report on the administration of an antiplatelet drug for Trousseau's syndrome, it may be effective if a blood clot is formed directly in an artery due to hypercoagulability. However, coadministration of heparin and antiplatelet drugs increase the risk of bleeding and should be done with caution. In this case, gastrointestinal bleeding occurred at the onset of myocardial infarction, so antiplatelet drugs could not be used in combination with heparin. Therefore, stent treatment with the catheter for myocardial infarction was not performed. In Trousseau's syndrome, it is important to evaluate the presence of intracardiac thrombus and patent foramen ovale with ultrasonography at the time of onset. If it is confirmed that they do not exist, hypercoagulation due to the tumor may have directly caused clots in arteries, and antiplatelet drugs may be effective. Although the prognosis of Trousseau's syndrome is generally poor, there is a possibility of improving the prognosis if the tumor can be reduced by treatment with chemotherapy. However, chemotherapy was ineffective in this case, and anticoagulation therapy with heparin did not prevent thrombotic exacerbation.

We experienced a case of Trousseau's syndrome with various thrombotic symptoms such as multiple cerebral infarctions, myocardial infarction, and pulmonary embolism due to ovarian cancer. Regardless of heparin administration, thrombus exacerbation could not be prevented. The mechanism of the onset of Trousseau's syndrome has not been determined yet, and it is hoped that future research will develop an effective treatment method.
